# Critical Appraisal of Anesthesiology Educational Research for 2017

**DOI:** 10.7759/cureus.4838

**Published:** 2019-06-05

**Authors:** Lara Zisblatt, Fei Chen, Dawn Dillman, Amy N DiLorenzo, Mark P MacEachern, Amy Miller Juve, Emily E Peoples, Ashley E Grantham

**Affiliations:** 1 Department of Anesthesiology, University of Michigan, Ann Arbor, USA; 2 Department of Anesthesiology, University of North Carolina School of Medicine, Chapel Hill, USA; 3 Department of Anesthesiology & Perioperative Medicine, Oregon Health & Science University, Portland, USA; 4 Department of Anesthesiology, University of Kentucky, Lexington, USA; 5 Taubman Health Sciences Library, University of Michigan, Ann Arbor, USA; 6 Department of Anesthesiology, Duke University, Durham, USA

**Keywords:** medical education, bibliometric, anesthesiology

## Abstract

Background

Critical appraisals provide a method for establishing the status of an area of study or evaluating the effectiveness of literature within it. The purpose of this study was to review and appraise studies published in 2017 on medical education in anesthesiology and to provide summaries of the highest-quality medical education research articles in the field.

Methods

Three Ovid MEDLINE databases, Embase.com, Education Resources Information Center (ERIC), and PsycINFO, were searched followed by a manual review of articles published in the highest impact factor journals in both the fields of anesthesiology and medical education. Abstracts were double-screened and quantitative articles subsequently scored by three randomly assigned raters. Qualitative studies were scored by two raters. Two different rubrics were used for scoring quantitative and qualitative studies, both allowed for scores ranging from 1-25.

Results

A total of 864 unique citations were identified through the search criteria. Of those, 62 articles met the inclusion criteria, with 59 quantitative and three qualitative. The top 10 papers with the highest scores were reported and summarized.

Discussion

As the first article to critically review the literature available for education in anesthesiology, we hope that this study will serve as the first manuscript in an annual series that will help individuals involved in anesthesiology education gain an understanding of the highest-quality research in the field. Once this process is repeated, trends can be tracked and serve as a resource to educators and researchers in anesthesiology for years to come.

## Introduction

The need for medical education research in the field of anesthesiology is vital. To ensure the best educational and clinical outcomes, medical education should be based on the best available evidence so that science can shape the nature of our practice [1]. Yet, in general, medical education research is underfunded [2] and the studies that do exist are often criticized for lacking rigor [3].

The purpose of this study is to review and appraise all of the studies published in 2017 on medical education in anesthesiology and to provide summaries of the highest-quality medical education research articles in the field. We assert that a regular, critical review of the literature in anesthesiology education will highlight rigorous research being performed in the field, reinforce best practices, and identify areas actively needing further investigation. In addition, synthesizing key findings for a time-pressed audience may foster the application of the knowledge gained from these studies to daily practice. Gaps in anesthesiology education research and literature may also be discovered through this critical review.

This study is based on a series of critical appraisals conducted over the last 10 years in emergency medicine (EM) with the purpose described as providing a “valuable resource for EM educators and researchers invested in the scholarship of teaching" [4]. Similarly, we hope that this will be the first article in a yearly series that will allow us to track the state of research in medical education in anesthesiology.

## Materials and methods

Article identification

To identify all articles in anesthesiology education, a medical librarian (MM) searched three Ovid MEDLINE databases (MEDLINE, In-Process & Other Non-Indexed Citations, Epub Ahead of Print), Embase.com, Education Resources Information Center (ERIC; via FirstSearch), and PsycINFO (via EBSCOhost). These databases were selected to cast a suitable net over the health sciences, education, and psychology literature. Each search consisted of a set of anesthesiology and education terms. Appropriate controlled terms were used in MEDLINE, Embase, and ERIC and supplemented with a search of article titles and abstract keywords. The PsycINFO search relied entirely on article title and abstract. All searches were initially run on January 30, 2018, and rerun on October 3, 2018, to allow time for studies published in 2017 to be indexed in each database. Animal and non-English studies were excluded from the search results, and all searches were limited to publication year 2017 with publications pre-printed in 2017 excluded. The Ovid MEDLINE search is available in Table 1.

**Table 1:** Database Search Used in Ovid MEDLINE

1	(exp anesthesiology/ or exp anesthetists/ or (anesthe* or anaesthe*).tw.) and (exp education/ or education.sh. or (academic* or class or classes or course* or curricul* or educat* or fellow or fellows or fellowship or instruct* or intern or interns or internship or learn or learner or learning or resident or residents or residenc* or school* or student* or teach* or train* or workshop*).ti.) and english.la. not (exp animals/ not humans/)
2	limit 1 to yr="2017"

All reproducible searches are included in the supplemental materials. Endnote X8 (Clarivate Analytics, Pennsylvania, United States) was used to remove duplicates.

Also, in November 2018, we conducted a manual review of the highest impact factor journals in both the fields of anesthesiology and medical education, as identified in Journal Citation Reports (Clarivate Analytics), to ensure that our searches did not exclude any relevant articles. For medical education, the list included Academic Medicine(Impact Factor: 4.801)*, *Medical Education(Impact Factor: 3.617)*, *Advances in Health Sciences Education(Impact Factor: 1.46)*, *Medical Teacher(Impact Factor: 2.450)*, *and Simulation in Healthcare* *(Impact Factor: 2.340)​​​​​​​. For anesthesiology, the list included Anesthesiology (Impact Factor: 5.163)​​​​​​​, Anesthesia & Analgesia (Impact Factor: 3.827)​​​​​​​, and British Journal of Anaesthesia (Impact Factor: 6.499)​​​​​​​. In this manual search, we also included the Journal of Education in Perioperative Medicine​​​​​​​ since it is the journal focused on medical education in anesthesiology.

Inclusion and exclusion criteria

We followed the same inclusion and exclusion criteria used by Heitz et al. in the critical appraisal of education in emergency medicine manuscript [3]. We included all levels of learners (students, residents/trainees, and practicing clinicians) and articles applicable to both physicians and nurses in the field of anesthesiology. Authors that applied and verified the inclusion criteria included both experts in anesthesiology education and anesthesiologists. Studies were defined as a) hypothesis-testing investigations, b) evaluations of education interventions, or c) explorations of educational problems. Publications were excluded if they were: a) not studies (editorials, commentaries); b) short reports that lacked enough information to be evaluated; c) not relevant to anesthesiology learners; d) single-site survey studies; or e) studies that examined outcomes limited to an expected learning effect without a comparison group. 

Data collection

To create the list of articles to be included in the critical appraisal, one author (LZ) reviewed all abstracts and applied the inclusion and exclusion criteria. Two additional authors (AG, FC) were each assigned half of the abstracts to independently apply the inclusion and exclusion criteria to their assigned abstracts. If the initial reviewer (LZ) and the second reviewer (AG or FC) were in agreement, then the article was excluded. Differences of opinion were reconciled by a third reviewer (AG or FC), who was not initially assigned the abstract. The list of articles and abstracts were maintained in a Microsoft Excel 2010 database (Microsoft Corporation, Washington, United States).

Scoring

The quantitative and qualitative scoring rubrics developed by Heitz et al. were used to score each article. We piloted the quantitative scoring rubric by having all authors review five randomly chosen papers from the list of included abstracts. Through a series of conference calls and email communications, the authors worked to create a shared mental model and notes were added to the scoring rubric to help maintain stable definitions for all criteria.

Each quantitative article that met inclusion criteria was randomly assigned to three authors, resulting in each author independently scoring 23 articles. Qualtrics (2019; Utah, US) was used to capture all scoring data, which then was exported into Excel 2010 for analysis. Mean scores were calculated through Excel 2010 and the articles with the top 10 mean scores were selected. Inter-rater reliability was assessed with an intraclass correlation coefficient using a one-way random-effect model in SPSS 25.0 (IBM Corp., Armonk, NY, US). Since this study did not involve human subjects, Institutional Review Board approval was not sought.

Two authors (AG, LZ), who have expertise in qualitative research methods, scored all qualitative articles. Each item was discussed and the two authors (AG, LZ) agreed upon scoring for each item.

Table 2 and Table 3 show the scoring rubrics used for the quantitative and qualitative articles, respectively.

**Table 2:** Quantitative Scoring Rubric

Domain	Item	Item score	Max score 25
Introduction (select all that apply)			3
	Appropriate description of background literature	1	
	Clearly frame the problem	1	
	Clear objective/hypothesis	1	
Measurement 1. Methodology (select one)			2
	Has no pre-test or post-test	1	
	Has a post-test only (If has a pre-test do NOT select)	1	
	has a pre-test and a post-test	2	
2. Groups (select all that apply)			2
	Both experimental and control group	1	
	Random assignment to groups	1	
Data Collection 1. Institutions (select one) Number of institutions refers to origin of study participants (not study authors)			2
	1 institution	0	
	2 institutions	1	
	3 or more institutions	2	
2. Response rate (select one) -Response rate is the proportion of those eligible who completed follow-up assessment. -Use "N/A" only if a response rate truly does not apply (e.g., data obtained from a medical record or professional organization database).			2
	< 50% or not reported:	0	
	50%–74%	1	
	≥ 75%	2	
	N/A	0	
Data analysis			
1. Appropriateness(select one) Considered “0” if there is statistical error or if authors failed to analyze data			1
	Data analysis inappropriate for study design/type of data	0	
	Data analysis appropriate for study design and type of data	1	
2. Sophistication (select all that apply) (Any test of statistical inference is considered “beyond descriptive.”)			2
	Descriptive analysis only	0	
	Beyond descriptive analysis	1	
	Includes power analysis	1	
Discussion (select all that apply)			3
	Data support conclusion	1	
	Conclusion clearly addresses hypothesis/objective	1	
	Conclusions placed in context of literature	1	
Limitations (select one)			2
	Limitations not identified accurately	0	
	Some limitations identified	1	
	Limitations well addressed	2	
Innovation of project (select one)			2
	Previously described methods	0	
	New use for known assessment/intervention	1	
	New assessment/intervention methodology	2	
Relevance of project (select one)			2
	Impractical to most programs	0	
	Relevant to some	1	
	Relevant to many programs	2	
Clarity of writing (select one)			2
	Unsatisfactory	0	
	Fair	1	
	Excellent	2	
Total			25

**Table 3:** Qualitative Scoring Rubric

Domain	Item		Item score	Max score
Introduction (select all that apply)				3
	Appropriate description of background literature		1	
	Clearly frame the problem		1	
	Clear objective/hypothesis		1	
Measurement				3
1. Methodology (select all that apply)				
	Appropriate for study question	1		
2. Sampling of participants (select all that apply)				
	Appropriate study population		1	
	Enrolled full range of cases/settings beyond convenience		1	
Data Collection 1. Institutions (select one) Number of institutions refers to origin of study participants (not study authors)				3
	1 institution		0	
	2 institutions		1	
	3 or more institutions		2	
2. Sample size determination (select one)				
	Appropriate sample size determination		1	
Data analysis (select all that apply)				5
	Clear, reproducible “audit trail” documenting systematic procedure for analysis		1	
	Data saturation through a systematic iterative process of analysis		1	
	Addressed contradictory responses		1	
	Incorporated validation strategies (e.g., member checking, triangulation)		1	
	Addressed reflexivity (impact of researcher’s background, position, biases on study)		1	
Discussion (select all that apply)				3
	Data support conclusion		1	
	Conclusion clearly addresses hypothesis/objective		1	
	Conclusions placed in context of literature		1	
Limitations (select one)				2
	Limitations not identified accurately		0	
	Some limitations identified		1	
	Limitations well addressed		2	
Innovation of project (select one)				2
	Previously described methods		0	
	New use for known assessment/intervention		1	
	New assessment/intervention methodology		2	
Relevance of project (select one)				2
	Impractical to most programs		0	
	Relevant to some		1	
	Relevant to many programs		2	
Clarity of writing (select one)				2
	Unsatisfactory		0	
	Fair		1	
	Excellent		2	
Total				25

Both rubrics allowed for scores ranging from 1-25, with the highest possible score set to 25 to make the scores comparable despite the difference in study type.

## Results

A total of 864 unique citations were identified through the search criteria. Of those, 62 articles met the inclusion criteria (59 quantitative and three qualitative; see the Appendix for the full list of articles included in the critical appraisal). The intraclass correlation coefficient (ICC) found an average measure of ICC(1) = 0.717 (95%CI = (0.549, 0.830)) for all quantitative study articles scored.

The mean score for all 59 quantitative articles included was 15.60 out of a possible 25 points, with the score for articles ranging from 6.67 to 21.33. The top 10 scored articles had a mean score of 20.43, with scores ranging from 19.33 to 21.33.

The average score for the qualitative papers was 5.38, with scores ranging from 2 to 8.5. The score of 19.33 was chosen as the threshold for inclusion in the top 10 since that was the lowest score for the top 10 quantitative papers, thus no qualitative papers were included.

Top 10 papers

An annotated bibliography of the top 10 papers is listed below in alphabetical order by first author.

1. Bloch A, Von Arx R, Etter R, Berger D, Kaiser H, Lenz A, Merz TM: Impact of simulator-based training in focused transesophageal echocardiography: a randomized controlled trial. Anesth Analg. 2017, 125:1140-1148 [5].

Description

Using a prospective, randomized controlled design with a blinded outcome assessment, this study aimed to determine the impact of simulator-based transesophageal echocardiography (TEE) training on the ability of novice operators to perform and interpret a focused critical care transesophageal echocardiography (TEE).

Significance

One major contribution of this work is the development of an exam-quality scoring tool that included the assessment of the quality of the images acquired as well as the interpretation of the images. There can be many applications of such a tool, including the assessment of learners, quality control for practicing clinicians, and further evaluation of training interventions.

2. Bong CL, Lee S, Ng ASB, Allen JC, Lim EHL, Vidyarthi A. The effects of active (hot-seat) versus observer roles during simulation-based training on stress levels and non-technical performance: a randomized trial. Adv Simul. 2017, 2:7 [6].

Description

This study compared stress levels and non-technical skills, measured by the Anesthetist’s Non-Technical Skills (ANTS) score, between trainees who were in the “hot-seat” role during simulation-based training as compared to those who were observers. The authors found that stress levels, measured via salivary cortisol, were lower for observers than hot-seat participants and that “observers of SBT [simulation-based training] achieved an equivalent level of non-technical performance.”

Significance

As the authors note, these findings have the potential to make simulation less resource-intensive for institutions to implement and to impact the design of simulation learning experiences. However, further work is needed to attempt to replicate these results in other settings.

3. Friedman Z, Perelman V, McLuckie D, Andrews M, Noble LM, Malavade A, Bould MD. Challenging authority during an emergency-the effect of a teaching intervention. Crit Care Med, 2017, 45:e814-e820 [7].

Description

This study looked at the impact of an educational intervention on the ability of residents to intervene when an incorrect decision that could impact patient safety was made by a superior during a simulated experience.

Significance

The hierarchical nature of healthcare makes it hard for trainees to challenge authority even when a clear mistake that can impact patient outcomes is about to occur. This study showed that a simple, low-cost educational intervention could improve the frequency and quality of a resident’s willingness and ability to challenge an incorrect patient care decision made by a superior.

4. Goldberg A, Samuelson S, Khelemsky Y, Katz D, Weinberg A, Levine A, Demaria S. Exposure to simulated mortality affects resident performance during assessment scenarios. Simul Healthc. 2017, 12:282-288 [8].

Description

Using a randomized design, this study sought primarily to determine whether there was a difference in performance for residents exposed to varying levels of simulated mortality during training scenarios. Residents in the variable death group had improved nontechnical skills while the always and never death groups showed no difference.

Significance

While mortality in simulation is still controversial, this study starts to show how the thoughtful use of mortality, when it is related to the performance of the learner, can improve nontechnical skills without causing higher levels of anxiety. This may help educators make more informed decisions about whether or not to include patient mortality in simulation.

5. Jullia M, Tronet A, Fraumar F, et al. Training in intraoperative handover and display of a checklist improve communication during transfer of care: an interventional cohort study of anaesthesia residents and nurse anaesthetists. Eur J Anaesthesiol, 2017, 34:471 [9].

Description

The authors showed that intraoperative handover training and display of a checklist in the OR improved the communication of residents and certified registered nurse anesthetists (CRNAs) during intraoperative transfers of anesthesia care.

Significance

With duty-hour restrictions came the potential increase in handovers among trainees. This study helps to address a gap in the standardization of intraoperative handovers through training and the creation of a checklist to improve communication. These themes have high generalizability, with the potential to reduce preventable adverse events. Future areas of study might explore the qualitative handover factors beyond the quantitative checklist items and may offer valuable insight into the retention and clarity of information transferred.

6. Katz D, Zerillo J, Kim S, Hill B, Wang R, Goldberg A, DeMaria S. Serious gaming for orthotopic liver transplant anesthesiology: a randomized control trial. Liver Transpl, 2017, 23:430-439 [10].

Description

This randomized control study showed that a serious game designed to teach orthotopic liver transplantation (OLT) anesthetic management improved resident performance in simulated orthotopic liver transplantation (OLT).

Significance

This study found adding a serious game to an existing educational curriculum was a feasible and cost-effective way to enhance learning in anesthesiology residents. The use of a serious game to enhance education can potentially be used for any topic in any field, making the findings widely applicable.

7. Kleiman AM, Forkin KT, Bechtel AJ, Collins SR, Ma JZ, Nemergut EC, Huffmyer JL. Generative retrieval improves learning and retention of cardiac anatomy using transesophageal echocardiography. Anesth Analg. 2017, 124:1440-1444 [11].

Description

This study showed that asking learners to guess (generative retrieval) the answers to questions before the answer was given helped them learn normal cardiovascular ultrasound anatomy through TEE images.

Significance

While this study focuses on learning TEE, the technique of generative retrieval could be used for any subject in anesthesiology and beyond. This can have implications to the way in which curricula are designed to allow learners the opportunity to guess even before they are taught new material.

8. Merry AF, Hannam JA, Webster CS, et al. Retesting the hypothesis of a clinical randomized controlled trial in a simulation environment to validate anesthesia simulation in error research (the VASER study). Anesthesiology. 2017, 126:472-481 [12].

Description

This study showed that a high-fidelity simulation-based study could be used to justify the same principal conclusions as a clinical study.

Significance

This study demonstrated the ability to apply simulation research to clinical settings when studies and the simulation experiences are carefully constructed. The authors suggest that studies on human factors, teamwork, and communication lend themselves particularly well to investigations using a simulated environment. Even though the study is about whether an intervention can be tested through simulation, the results also support the connection between simulation and real life, which has implications for the use of simulation in training.

9. Saddawi-Konefka D, Baker K, Guarino A, Burns SM, Oettingen G, Gollwitzer PM, Charnin JE. Changing resident physician studying behaviors: a randomized, comparative effectiveness trial of goal setting versus use of WOOP. J Grad Med Educ. 2017, 9:451-457 [13].

Description

The purpose of this study was to evaluate WOOP (Wish, Outcome, Obstacle, Plan), a validated tool for improving learner self-regulation as a means of improving study habits in residents on an intensive care unit (ICU) rotation.

Significance

The WOOP is a free and easily used self-regulation tool that this study shows to have potential to help resident learners. The application of the principles of cognitive psychology to education is a frontier for medical education. Future areas of investigation could include using the WOOP in rotations with potentially less well-defined content (i.e. general OR rotations) or evaluating other tools to improve self-regulation.

10. Spadaro S, Karbing DS, Fogagnolo A, et al. Simulation training for residents focused on mechanical ventilation: a randomized trial using mannequin-based versus computer-based simulation. Simul Healthc. (2017), 12:349 [14].

Description

This study compared two strategies (mannequin- and computer-based simulation modalities) for teaching lung-protective ventilation strategies with low tidal volume to anesthesiology residents. The authors found that “mannequin-based simulation seemed more effective than computer-based simulation for improving knowledge and skills related to mechanical ventilation.”

Significance

This study provides a methodologically rigorous model for assessing varying modalities of simulation training. Further, it offers insight into training models for mechanical ventilation.

## Discussion

To our knowledge, this manuscript is the first to critically review anesthesiology education literature with the goal of quantitatively and qualitatively assessing studies for scientific rigor and academic and clinical merit. We envision this manuscript as the first annual installment to help practitioners better understand the state of research in the field and contribute to the increased application of evidence-based practices in anesthesiology education.

Since this is only the first review of its kind, we cannot establish trends over time; however, there were a few commonalities among the studies we reviewed that are of note. First, looking at the scores in each category included on the rubric for quantitative articles, less than 25% (n=15) of articles included a control group, less than 20% (12) included random assignment, and only 24% (15) included power analysis. This shows a majority of the articles reviewed lacked basic rigor. While innovative concepts might require piloting and sometimes less rigorous methodology to establish feasibility, only 23% (14) of articles were scored as an innovative assessment or intervention. This is further evidence that supports the concerns about the rigor of medical education research [3]. In addition, none of the very few qualitative articles achieved a score high enough to be included in our top list. Since medical education research is trying to build on our understanding of how and why things work, qualitative research could help with the fundamental exploration needed to answer these questions.

While great care was taken to ensure rigor in this appraisal, this study is not without limitations. Even though rigorous search methods were applied to locate articles relevant to anesthesiology education, the searches may have erroneously omitted or excluded some articles that should have been included. Particularly susceptible to this type of omission are those articles published in a journal where the focus is on a field outside of anesthesiology or medical education. However, the top 10 articles come from nine different journals showing variety among the journals represented. In addition, a total of 39 different journals were represented by the 63 articles included in the critical appraisal review.

Another potential limitation is the nature of the rating process and the assessment tools. Even though we did rater training and worked to stabilize the definitions for each criterion included in the rubric, there were elements that were subject to interpretation and may have resulted in differences in scores. However, since the judgment of the reviewers is inherent to the process of a critical appraisal, some bias is inherent to the process. Nonetheless, there was high inter-rater reliability of our assessment, considering ICC(1) values tend to be very low.

In addition, the allocation of points within the quantitative study scoring rubric favored studies that included an educational intervention. This systematic bias in the scoring instrument left some high-quality articles of non-intervention studies with low scores. For example, the Baker et al. study [15] examining retaliation in faculty and trainee evaluations is highly relevant to anesthesiology education and had a sample size of over 25,000 evaluations. However, it lost points for not having a control group, not using a pre-/post-model, and only including one institution while other studies with a very small sample size that included those elements scored higher.

As previously stated, we hope to continue this initiative on an annual basis. To better ensure that the highest-quality studies are being highlighted, regardless of the type of study design or methodology chosen, we aim to develop a refined rubric to mitigate our identified limitations.

## Conclusions

As the first article to critically review the literature available for education in anesthesiology, we hope that this study will serve as the first manuscript in an annual series that will help individuals involved in anesthesiology education gain an understanding of the highest-quality research in the field. Once this process is repeated, trends can be tracked and serve as a resource to educators and researchers in anesthesiology for years to come.

## Appendices

**Table 4:** Full List of Articles Included in the Critical Appraisal

Article #	First Author	Title	Journal	Type
1	Artyomenko, VV	Anaesthesiologists' simulation training during emergencies in obstetrics	Romanian Journal of Anaesthesia & Intensive Care	Quantitative
2	Baker, K	A Feedback and Evaluation System That Provokes Minimal Retaliation by Trainees	Anesthesiology	Quantitative
3	Bakshi, SG	Role of WhatsApp-based discussions in improving residents' knowledge of post-operative pain management: a pilot study	Korean Journal of Anesthesiology	Quantitative
4	Bick, JS	Standard Setting for Clinical Performance of Basic Perioperative Transesophageal Echocardiography: Moving beyond the Written Test	Anesthesiology	Quantitative
5	Bloch, A	Impact of Simulator-Based Training in Focused Transesophageal Echocardiography: A Randomized Controlled Trial	Anesthesia & Analgesia	Quantitative
6	Bong, CL	The effects of active (hot-seat) versus observer roles during simulation-based training on stress levels and non-technical performance: a randomized trial	Adv Simul	Quantitative
7	Bracco, F	Adaptation of non-technical skills behavioural markers for delivery room simulation	BMC Pregnancy & Childbirth	Quantitative
8	Castanelli, DJ	Measuring the anaesthesia clinical learning environment at the department level is feasible and reliable	British Journal of Anaesthesia	Quantitative
9	Cole, DC	Resident Physicians Improve Nontechnical Skills When on Operating Room Management and Leadership Rotation	Anesthesia & Analgesia	Quantitative
10	Copson, S	The effect of a multidisciplinary obstetric emergency team training program, the In Time course, on diagnosis to delivery interval following umbilical cord prolapse - A retrospective cohort study	Australian & New Zealand Journal of Obstetrics & Gynaecology	Quantitative
11	Corvetto, MA	Validation of the imperial college surgical assessment device for spinal anesthesia	BMC Anesthesiology	Quantitative
12	Crane, MF	Positive Affect Is Associated With Reduced Fixation in a Realistic Medical Simulation	Hum Factors	Quantitative
13	Dexter, F	Content analysis of resident evaluations of faculty anesthesiologists: supervision encompasses some attributes of the professionalism core competency	Canadian Journal of Anaesthesia	Qualitative
14	Dexter, F	With directed study before a 4-day operating room management course, trust in the content did not change progressively during the classroom time	Journal of Clinical Anesthesia	Quantitative
15	Dexter, F	Measurement of faculty anesthesiologists' quality of clinical supervision has greater reliability when controlling for the leniency of the rating anesthesia resident: a retrospective cohort study	Canadian Journal of Anaesthesia	Quantitative
16	DuCanto, J	Novel Airway Training Tool that Simulates Vomiting: Suction-Assisted Laryngoscopy Assisted Decontamination (SALAD) System	The Western Journal of Emergency Medicine	Quantitative
17	Easdown, LJ	A Checklist to Help Faculty Assess ACGME Milestones in a Video-Recorded OSCE	J Grad Med Educ	Quantitative
18	Eloy, JD	Fellowships Represent a Logical Target for Cultivating Research in Academic Anesthesiology	J Educ Perioper Med	Quantitative
19	Ergun, S	Mentorship in anesthesia: a survey of perspectives among Canadian anesthesia residents	Canadian Journal of Anaesthesia	Quantitative
20	Evain, JN	Residual anxiety after high fidelity simulation in anaesthesiology: An observational, prospective, pilot study	Anaesthesia Critical Care and Pain Medicine	Quantitative
21	Friedman, Z	Challenging authority during an emergency - The effect of a teaching intervention	Critical Care Medicine	Quantitative
22	Geeraerts, T	Physiological and self-assessed psychological stress induced by a high fidelity simulation course among third year anesthesia and critical care residents: An observational study	Anaesthesia Critical Care & Pain Medicine	Quantitative
23	Goldberg, A	Exposure to Simulated Mortality Affects Resident Performance During Assessment Scenarios	Simulation in Healthcare: The Journal of The Society for Medical Simulation	Quantitative
24	Gouin, A	Evolution of stress in anaesthesia registrars with repeated simulated courses: An observational study	Anaesthesia Critical Care & Pain Medicine	Quantitative
25	Gupta, R	Career Development Guidance and Mentorship during Anesthesia Residency Training: An Internet Survey	The Journal of Education in Perioperative Medicine	Quantitative
26	Harvey, R	The impact of didactic read-aloud action cards on the performance of cannula cricothyroidotomy in a simulated ‘can't intubate can't oxygenate’– scenario	Anaesthesia	Quantitative
27	Heck, MC	An Evaluation of CA-1 Residents' Adherence to a Standardized Handoff Checklist	The Journal of Education in Perioperative Medicine	Quantitative
28	Howe, PW	A qualitative exploration of anesthesia trainees' experiences during transition to a children's hospital	Paediatric anaesthesia	Qualitative
29	Isaak, RS	A Descriptive Survey of Anesthesiology Residency Simulation Programs: How Are Programs Preparing Residents for the New American Board of Anesthesiology APPLIED Certification Examination?	Anesthesia & Analgesia	Quantitative
30	Isaranuwatchai, W	A cost-effectiveness analysis of self-debriefing versus instructor debriefing for simulated crises in perioperative medicine in Canada	Journal of Educational Evaluation for Health Professions	Quantitative
31	Jirativanont, T	Validity evidence of non-technical skills assessment instruments in simulated anaesthesia crisis management	Anaesthesia & Intensive Care	Quantitative
32	Jullia, M	Training in intraoperative handover and display of a checklist improve communication during transfer of care: An interventional cohort study of anaesthesia residents and nurse anaesthetists	European journal of anaesthesiology	Quantitative
33	Katz, D	Serious gaming for orthotopic liver transplant anesthesiology: A randomized control trial	Liver Transplantation	Quantitative
34	Kaur, G	Global health education in United States anesthesiology residency programs: a survey of resident opportunities and program director attitudes	BMC Medical Education	Quantitative
35	Kimatian, S	Undirected learning styles and academic risk: Analysis of the impact of stress, strain and coping	J Educ Perioper Med	Quantitative
36	Kleiman, AM	Generative Retrieval Improves Learning and Retention of Cardiac Anatomy Using Transesophageal Echocardiography	Anesthesia & Analgesia	Quantitative
37	Lean, LL	End-task versus in-task feedback to increase procedural learning retention during spinal anaesthesia training of novices	Advances in Health Sciences Education	Quantitative
38	Lockman, JL	Working to define professionalism in pediatric anesthesiology: a qualitative study of domains of the expert pediatric anesthesiologist as valued by interdisciplinary stakeholders	Paediatric Anaesthesia	Qualitative
39	Marchalot, A	Effectiveness of a blended learning course and flipped classroom in first year anaesthesia training	Anaesthesia Critical Care & Pain Medicine	Quantitative
40	Martinelli, SM	Results of a Flipped Classroom Teaching Approach in Anesthesiology Residents	Journal of Graduate Medical Education	Quantitative
41	Mehta, KH	Developing competency in post-graduate students of anaesthesiology for taking informed consent for elective caesarean section	Indian Journal of Anaesthesia	Quantitative
42	Merry, AF	Retesting the Hypothesis of a Clinical Randomized Controlled Trial in a Simulation Environment to Validate Anesthesia Simulation in Error Research (the VASER Study)	Anesthesiology	Quantitative
43	Mok, D	Point-of-care ultrasonography in Canadian anesthesiology residency programs: a national survey of program directors	Canadian Journal of Anaesthesia	Quantitative
44	Neal, JM	Regional Anesthesia and Pain Medicine: US Anesthesiology Resident Training-The Year 2015	Regional Anesthesia & Pain Medicine	Quantitative
45	Ortega, R	An innovative textbook: design and implementation	Clin Teach	Quantitative
46	O'Shaughnessy, SM	First Year Specialist Anaesthesia Training in Ireland: A Logbook Analysis	International Journal of Higher Education	Quantitative
47	Ozcan, ATD	Comparison of endotracheal tube cuff pressure values before and after training seminar	Journal of Clinical Monitoring & Computing	Quantitative
48	Pelloux, S	Peripheral venous catheter insertion simulation training: A randomized controlled trial comparing performance after instructor-led teaching versus peer-assisted learning	Anaesthesia Critical Care & Pain Medicine	Quantitative
49	Prin, M	International Elective Opportunities in United States Anesthesia Residency Programs	J Educ Perioper Med	Quantitative
50	Rinehart, J	Anesthesiology Residency Curriculum and Implementation of a Perioperative Surgical Home Curriculum: A Survey Study	J Educ Perioper Med	Quantitative
51	Robertson, AC	Using the Teaching Perspectives Inventory as an Introduction to a Residents-as-Teachers Curriculum	J Educ Perioper Med	Quantitative
52	Saddawi-Konefka, D	Changing Resident Physician Studying Behaviors: A Randomized, Comparative Effectiveness Trial of Goal Setting Versus Use of WOOP	Journal of Graduate Medical Education	Quantitative
53	Scott-Herring, M	Development, Implementation, and Evaluation of a Certified Registered Nurse Anesthetist Preceptorship-Mentorship Program	J Contin Educ Nurs	Quantitative
54	Sidi, A	Simulation-Based Assessment Identifies Longitudinal Changes in Cognitive Skills in an Anesthesiology Residency Training Program	Journal of patient safety	Quantitative
55	Spadaro, S	Simulation Training for Residents Focused on Mechanical Ventilation: A Randomized Trial Using Mannequin-Based Versus Computer-Based Simulation	Simulation in Healthcare	Quantitative
56	Stone, L	Point-of-contact assessment of nurse anesthetists' knowledge and perceptions of management of anesthesia-related critical incidents^59^	AANA Journal	Quantitative
57	Vasian, HN	Anaesthesia and Intensive Care Residents' Perception of Simulation Training in Four Romanian Centres	The Journal of Critical Care Medicine	Quantitative
58	Watkins, SC	Evaluation of a Simpler Tool to Assess Nontechnical Skills During Simulated Critical Events	Simulation in Healthcare	Quantitative
59	Weller, JM	Making robust assessments of specialist trainees' workplace performance	British Journal of Anaesthesia	Quantitative
60	Wen, L	Encouraging Mindfulness in Medical House Staff via Smartphone App: A Pilot Study	Acad Psychiatry	Quantitative
61	Zhou, Y	Effect of the BASIC Examination on Knowledge Acquisition during Anesthesiology Residency	Anesthesiology	Quantitative
62	Zhou, Y	Effectiveness of Written and Oral Specialty Certification Examinations to Predict Actions against the Medical Licenses of Anesthesiologists	Anesthesiology	Quantitative
